# Longitudinal Associations Between Cumulative Physical Activity and Change in Structure and Function of the Left Side of the Heart: The Tromsø Study 2007–2016

**DOI:** 10.3389/fcvm.2022.882077

**Published:** 2022-05-12

**Authors:** Kim Arne Heitmann, Boye Welde, Maja-Lisa Løchen, Michael Stylidis, Henrik Schirmer, Bente Morseth

**Affiliations:** ^1^School of Sport Sciences, UiT The Arctic University of Norway, Tromsø, Norway; ^2^Centre for Research and Education, University Hospital of Northern Norway, Tromsø, Norway; ^3^Department of Community Medicine, UiT The Arctic University of Norway, Tromsø, Norway; ^4^Department of Cardiology, Akershus University Hospital, Lørenskog, Norway; ^5^Institute of Clinical Medicine, University of Oslo, Oslo, Norway; ^6^Department of Clinical Medicine, UiT The Arctic University of Norway, Tromsø, Norway

**Keywords:** athlete's heart, cardiac, echocardiography, ejection fraction, exercise, left atrium, left ventricle, public health

## Abstract

**Background:**

Current knowledge about the relationship between physical activity (PA) and cardiac remodeling is mainly derived from cross-sectional studies of athletes, and there is a knowledge gap of this association in the general adult and elderly population. Therefore, we aimed to explore the longitudinal association between cumulative PA and change in cardiac structure and function in a general adult and elderly population.

**Methods:**

This longitudinal study includes 594 participants from the sixth (Tromsø6, 2007–08) and seventh (Tromsø7, 2015–16) survey of the Tromsø Study. Cardiac structure and function were assessed by echocardiography at two time points, and PA was self-reported by questionnaire at both time points. PA volume was expressed as cumulative PA (Low, Moderate, and Hard) and the association with left atrial (LA) and left ventricular (LV) structure and function was assessed using ANCOVA.

**Results:**

Overall, LA diameter index (LADi) increased significantly more in Hard compared to Moderate PA (+0.08 cm/m^2^, 95% CI 0.01–0.15, *p* = 0.020) from Tromsø6 to Tromsø7. When stratified by sex or age, higher levels of cumulative PA were associated with increased LADi in males and in participants <65 years only. Indexed LV mass (LVMi) increased significantly more in Moderate than in Low PA (+3.9 g/m^2.7^, 95% CI 0.23–7.57, *p* = 0.037). When stratified by sex or age, these changes in LVMi and indexed LV diameter (LVDi) were only significant in females. No significant associations were observed between cumulative PA and change in relative wall thickness, E/e' ratio, e' velocity, LV ejection fraction, and LADi/LVDi ratio.

**Conclusion:**

Higher levels of cumulative PA were associated with increased LADi in males and participants <65 years, and with increased LVMi and LVDi in females. Despite cardiac chamber enlargement, the pump function of the heart did not change with higher levels of PA, and the atrioventricular ratio was unchanged. Our results indicate that cardiac chamber enlargement is a physiological response to PA.

## Introduction

Changes in cardiac structure and function can occur as a result of physiological remodeling from exercise or pathological remodeling (1). Whereas, physiological remodeling is considered benign adaptations, pathological remodeling is associated with increased risk of cardiovascular diseases and mortality. Both left atrial (LA) enlargement and left ventricular (LV) hypertrophy are independent risk factors for cardiovascular morbidity and mortality (2–4) in the general population, and occurs in response to risk factors such as hypertension, diabetes mellitus, and obesity via mechanisms such as increased pressure and volume overload (5).

Exercise-induced cardiac remodeling is generally considered a benign physiological adaption of exercise, and is characterized by enlarged cardiac chambers and increased LV wall thickness (6). Paradoxically, exercise-induced cardiac remodeling may mimic pathological remodeling (7, 8), and elite endurance athletes may have cardiac chamber size that overlap the size seen in cardiac pathology (7, 9, 10). However, it is observed that LA function (11, 12) and LV diastolic function (13, 14) are preserved in dynamic sport-elite athletes with LA enlargement, as well as in athletes with LV enlargement (7, 15).

Current knowledge about the relationship between physical activity (PA) and cardiac remodeling is mainly derived from cross-sectional studies of athletes (16), and there is a knowledge gap of this association in the general adult and elderly population. Hence, as most studies investigating the relationship between exercise and cardiac remodeling are cross-sectional, more longitudinal studies are needed. Therefore, our main objective was to explore the longitudinal association between cumulative PA and change in cardiac structure and function in a general adult and elderly population.

## Materials and Methods

### Study Population

The Tromsø Study is a single-center population-based cohort study with seven repeated health surveys of the population of the Tromsø municipality, Norway (17). This study includes participants from the sixth (Tromsø6, 2007–08) and seventh (Tromsø7, 2015–16) survey of the Tromsø Study.

**Figure 1:**
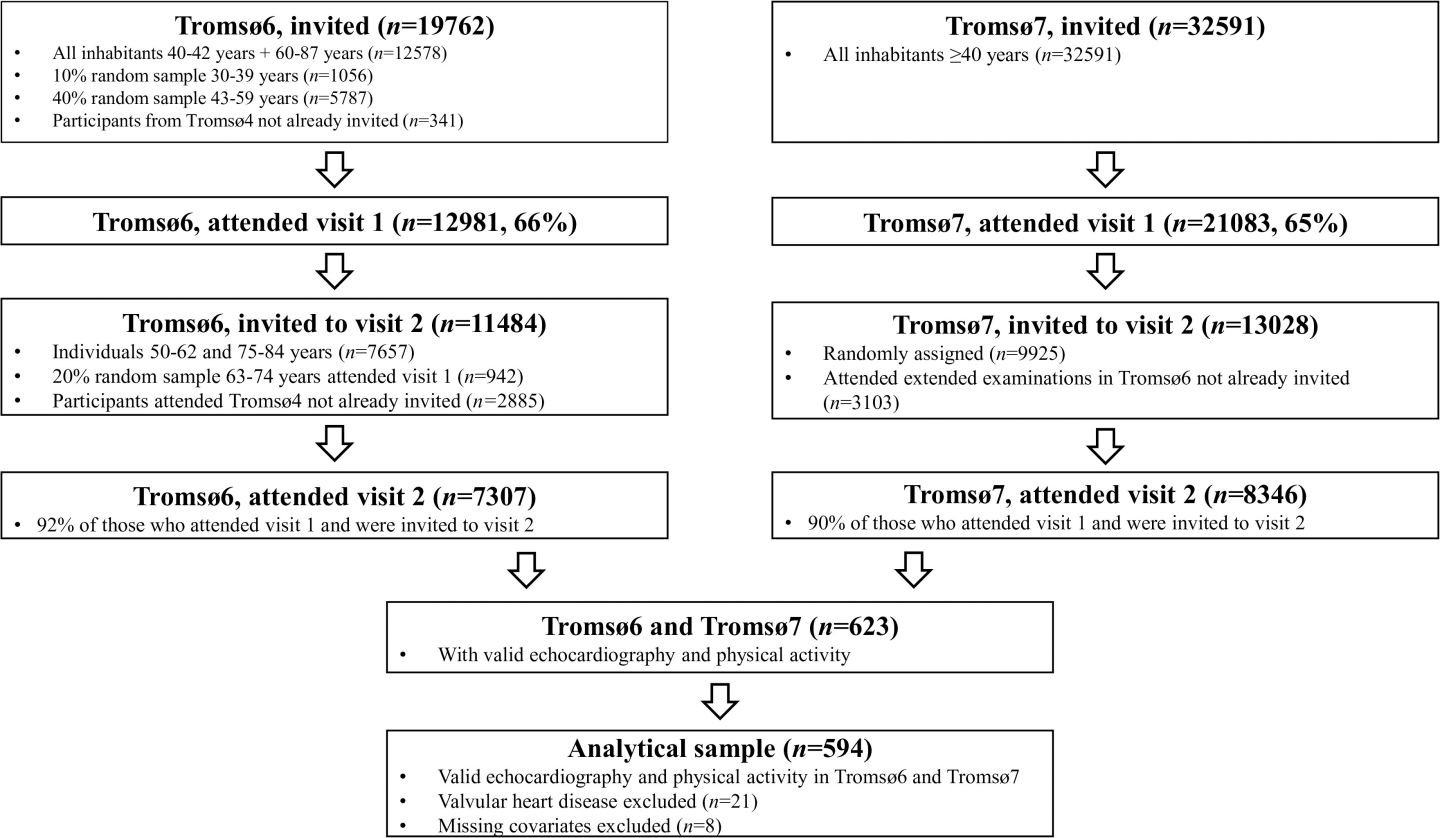
Flowchart over inclusion of participants: The Tromsø Study 2007–2016.

In total, 623 participants provided valid data on self-reported PA in combination with valid echocardiography data from Tromsø6 and Tromsø7. We excluded participants with valvular heart disease at baseline (*n* = 21). Furthermore, eight participants were excluded due to missing data on the covariate hypertension. Finally, our analytical sample consisted of 594 participants free from valvular heart disease, and with valid data on PA, echocardiography, and covariates at baseline (Figure 1). However, the number of participants differed slightly between the different analyses due to missing images and/or due to images with inappropriate quality.

### Physical Activity

**Figure 2:**
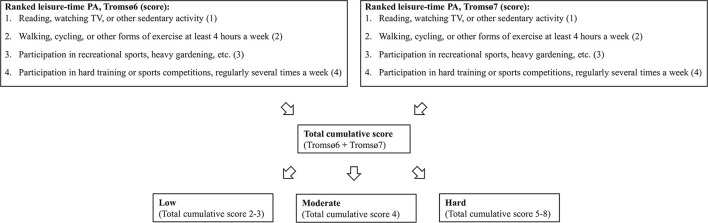
Overview of summation of PA from Tromsø6 and Tromsø7 into cumulative PA categories: The Tromsø Study 2007–2016. PA: physical activity.

PA was assessed using the Saltin-Grimby Physical Activity Level Scale (18), where the participants rank their leisure-time PA on a four-level scale. In our study, we summed up the participants' ranked leisure-time PA in Tromsø6 and Tromsø7 and combined them in a total cumulative score (Figure 2). Furthermore, we divided the cumulative score into three cumulative PA categories: (1) Low (total score 2–3), (2) Moderate (total score 4), and (3) Hard PA (total score 5-8).

### Cardiac Structure and Function

Echocardiographic examinations in Tromsø6 and Tromsø7 were performed by two qualified sonographers, for Tromsø6 collected from October 2007 to December 2008, using an Acuson Sequoia C512 (Acuson, Mountain View, California, USA) ultrasound scanner and for Tromsø 7 collected from August 2015 to October 2016 using a GE Vivid E9 (GE Medical, Horten, Norway) ultrasound scanner. The echocardiographic assessments were performed with the use of standard imaging planes in the left lateral decubitus position according to the joint American and European guidelines (19). In Tromsø6, the echocardiographic measurements were performed online in one heart cycle but remeasured if deviating from eye-balled estimates. In Tromsø7, the echocardiographic measurements were performed off-line on 3–5 consecutive cardiac cycles by a physician experienced in echocardiography (co-author MS), and the average was used in the analysis.

Cardiac dimensions were measured by M-mode echocardiography in the parasternal short axis view at the aortic valve level, after alignment of left ventricle in long axis view, according to the leading edge-to-leading edge convention (19). LV internal dimensions were measured at the end of diastole and systole and indexed to body surface area (LVDi) as cm/m^2^ (20). LA anteroposterior diameter was measured at the end of the LV systole and indexed to body surface area (LADi) as cm/m^2^. LA volume was measured at the end of the LV systole and calculated using the Simpson's biplane method from the apical four- and two chamber views and indexed to body surface area as mL/m^2^. LADi/LVDi ratio was calculated. Relative wall thickness was calculated with the formula (2 x posterior wall thickness) / (LV internal end-diastolic diameter). LV myocardial mass was calculated according to the cube formula (19), and further indexed to height by raising height to the power of 2.7 (LVMi), and are presented as g/m^2.7^ (21). LV ejection fraction (LV EF) was calculated using the Teichholz formula (22).

All Doppler examinations were performed in apical four-chamber view according to current recommendations (23). Mitral valve Doppler measurements were performed with a 2 mm Doppler sample volume placed between the mitral leaflet tips. Tissue Doppler measurements were performed with a 5 mm Doppler sample volume located at the septal and lateral side of the mitral annulus. Measurement of peak flow velocity in early diastole (E-wave) was measured with pulsed Doppler. Mitral annular e' velocity was measured with pulsed-wave tissue Doppler in both lateral and septal basal regions and furthermore averaged. E/e'ratio was calculated. Valvular heart disease was defined by the following criteria: (a) aortic stenosis (aortic valve mean gradient ≥15 mmHg) by continuous Doppler (24), (b) presence of mitral or (c) aortic regurgitation detected by color Doppler imaging with mitral insufficiency graded according to regurgitant jet area >4 cm^2^ (25), and aortic regurgitation graded by vena contracta width by color M-mode divided by of LV outflow tract diameter (>30% graded as moderate or higher) (25), and/or (d) mitral stenosis (E-wave deceleration time >350 msec and mitral E-wave >1 m/s) by pulsed Doppler (26). However, mitral stenosis was not identified in any subjects in our analytical sample.

In Tromsø7, an intra- and inter-observer study was performed on the echocardiography data (27). Intra-class correlation coefficients on Doppler indices and linear measurements were 0.90–0.99 in the intra-observer study and 0.84–0.98 in the inter-observer study. In Tromsø6, intra- and inter-observer variability on Doppler indices was evaluated by Bland-Altman analysis (24). The results showed mean inter-observer differences (95% limits of agreement) in the mean aortic gradient of −0.06 mmHg (−3.06 to 3.18). Intra-observer analysis gave a mean difference of −0.04 mmHg (−1.86 to 1.78) and 0.30 mm Hg (−3.96 to 4.56), respectively, in the two observers.

### Covariates

Details about collection of baseline data are described elsewhere, and all data were collected by specially trained research technicians (28). Baseline data from Tromsø6 include the following covariates extracted from self-reported questionnaires, physical examinations, and blood samples: Daily smoking (yes or previously/never), diabetes (yes/no), use of antihypertensives (currently or previously/never), myocardial infarction (previously/no), stroke (previously/no). Alcohol consumption was the product of two questions, one reporting number of units of alcohol and one reporting frequency of drinking.

Blood pressure was recorded three times with 1 min intervals after 2 min seated rest with an automatic device (Dinamap Pro care 300 Monitor, GE Healthcare, Oslo, Norway), the average from reading two and three was used in our analyses. Blood pressure was classified into hypertension groups (21): (a) Normotensive (systolic blood pressure <140 mmHg, diastolic blood pressure <90 mmHg, and no self-reported use of antihypertensives), (b) hypertensive, controlled (systolic blood pressure <140 mmHg, diastolic blood pressure <90 mmHg, and self-reported use of antihypertensives), (c) hypertensive, uncontrolled (systolic blood pressure ≥140 mmHg and/or diastolic blood pressure ≥90 mmHg, and self-reported use of antihypertensives), or d) hypertension, untreated (systolic blood pressure ≥140 mmHg and/or diastolic blood pressure ≥90 mmHg, and no self-reported use of antihypertensives). Height and weight were measured to the nearest decimal with participants wearing light clothing and no footwear. Body mass index was calculated as weight (kg) divided by height squared (*m*^2^).

Data on atrial fibrillation was derived from the diagnosis registry of the University Hospital of North Norway, the only hospital in the region, by linking the hospitals records of atrial fibrillation to the participants' unique Norwegian national 11-digit identification number (29). Blood samples were analyzed for low-density lipoprotein (LDL) cholesterol at the Department of Clinical Chemistry, University Hospital of North Norway.

### Statistical Methods

Descriptive characteristics of the study population are presented as means with standard deviations (SD) or percentages with number of observations (*n*). The associations between cumulative PA and cardiac structure and function were evaluated by one-way analysis of covariance with Bonferroni adjusted *post hoc* comparisons. Data are presented as adjusted means with standard error (SE), and effects with 95% confidence intervals (CI) unless otherwise stated. Model 1 is unadjusted, model 2 is adjusted for age, sex, body mass index, and hypertension groups.

Sex^*^PA was a significant interaction term in the association between PA and change in LA diameter (*p* = 0.024), body mass index^*^PA was a significant interaction term between PA and change in E/e' ratio (*p* = 0.028), and hypertension (normotensive/hypertensive)^*^PA was a significant interaction term between PA and change in average e' (*p* = 0.040).

To test the robustness of the fully adjusted model 2, we performed sensitivity analyses excluding participants with known cardiac pathologies (atrial fibrillation, myocardial infarction, stroke, and LV EF <40%) stepwise. Moreover, we performed sensitivity analysis adjusted for additional covariates (smoking, alcohol consumption, diabetes, and LDL cholesterol) stepwise added to the model.

For sensitivity analyses of the PA assessments, we compared the mean PA score in Tromsø6 with the mean PA score in Tromsø7, stratified by level of cumulative PA (Supplementary Table S1), to assess whether there were differences in PA score between Tromsø6 and Tromsø7 within each level of cumulative PA. Moreover, the activity level within each level of cumulative PA was quantified with accelerometry-measured PA, assessed by a triaxial accelerometer (wGT3X-BT, ActiGraph LLC, Pensacola, FL, USA), in Tromsø7 (Supplementary Table S2).

All statistical analyses were performed using SPSS version 28 (SPSS Inc., IL, USA), with a two-sided alpha ≤ 0.05 considered statistically significant.

## Results

In total, 266 males (61.1 ± 8.9 years) and 328 females (58.9 ± 9.9 years), ranging from 37 to 76 years, were included in our study. Descriptive baseline characteristics of the analytical sample, stratified by PA, is given in Table 1.

**Table 1:** Descriptive baseline characteristics stratified by level of cumulative physical activity: The Tromsø Study 2007–2008.

	**Low PA** **(*n* = 140)**	**Moderate PA** **(*n* = 259)**	**Hard PA** **(*n* = 195)**	**Total** **(*n* = 594)**
Age, years	61.4 (9.0)	59.2 (9.4)	59.9 (10.0)	60.0 (9.6)
Sex, % (*n*) female	60.7 (85)	62.9 (163)	41.0 (80)	55.2 (328)
Body mass index kg/m^2^	28.1 (4.6)	26.3 (3.7)	26.3 (3.6)	26.7 (4.0)
Systolic blood pressure, mmHg	141.1 (21.0)	136.6 (20.9)	136.6 (22.7)	137.7 (21.6)
Diastolic blood pressure, mmHg	78.1 (10.7)	78.0 (9.7)	78.4 (10.5)	78.2 (10.2)
LDL cholesterol, mmol/L	3.7 (1.0)	3.6 (1.1)	3.6 (0.9)	3.6 (1.0)
Hypertension, controlled, % (*n*)	11.4 (16)	6.2 (16)	6.2 (12)	7.4 (44)
Hypertension, uncontrolled, % (*n*)	17.9 (25)	11.6 (30)	15.4 (30)	14.3 (85)
Hypertension, untreated, % (*n*)	32.1 (45)	34.7 (90)	29.7 (58)	32.5 (193)
Myocardial infarction, %	5.8 (8)	5.0 (13)	3.7 (7)	4.8 (28)
Stroke, %	2.9 (4)	1.2 (3)	2.1 (4)	1.9 (11)
Atrial fibrillation, %	2.9 (4)	1.2 (3)	2.6 (5)	2.0 (12)
Diabetes, %	8.7 (12)	2.7 (7)	3.7 (7)	4.4 (26)
Smoking daily, % (*n*)	23.9 (33)	18.1 (47)	8.8 (17)	16.4 (97)
Alcohol, units/month	9.8 (13.6)	9.6 (11.1)	10.8 (11.6)	10.1 (11.9)
Echocardiography				
LV mass, g	176.5 (54.9)	155.1 (44.9)	175.2 (55.4)	166.7 (51.9)
LV mass, g female	156.3 (40.5)	132.2 (28.7)	138.8 (33.7)	139.9 (34.6)
LV mass, g male	207.3 (59.9)	194.0 (40.4)	200.8 (53.5)	199.7 (50.7)
LV mass index, g/h^2.7^	42.8 (12.2)	37.5 (8.7)	40.9 (11.5)	39.8 (10.8)
LV mass index, g/h^2.7^ female	41.6 (12.0)	35.2 (7.7)	36.7 (9.9)	37.2 (9.8)
LV mass index, g/h^2.7^ male	44.5 (12.4)	41.3 (9.0)	43.8 (11.7)	43.1 (11.0)
LA diameter, cm	3.8 (0.5)	3.6 (0.5)	3.8 (0.5)	3.7 (0.5)
LA diameter, cm female	3.6 (0.5)	3.4 (0.4)	3.6 (0.4)	3.5 (0.4)
LA diameter, cm male	4.0 (0.4)	4.0 (0.5)	4.0 (0.6)	4.0 (0.5)
LA diameter index, cm/m^2^	2.0 (0.2)	2.0 (0.2)	2.0 (0.3)	2.0 (0.2)
LA diameter index, cm/m^2^ female	2.0 (0.3)	2.0 (0.2)	2.0 (0.2)	2.0 (0.2)
LA diameter index, cm/m^2^ male	2.0 (0.2)	2.0 (0.2)	2.0 (0.3)	2.0 (0.3)
LV diameter, cm	5.1 (0.5)	5.0 (0.5)	5.2 (0.5)	5.1 (0.5)
LV diameter, cm female	5.0 (0.5)	4.9 (0.4)	4.9 (0.4)	4.9 (0.4)
LV diameter, cm male	5.3 (0.5)	5.3 (0.5)	5.4 (0.5)	5.3 (0.5)
LV diameter index, cm/m^2^	2.7 (0.3)	2.7 (0.3)	2.8 (0.2)	2.7 (0.3)
LV diameter index, cm/m^2^ female	2.8 (0.3)	2.8 (0.3)	2.8 (0.2)	2.8 (0.3)
LV diameter index, cm/m^2^ male	2.6 (0.2)	2.6 (0.3)	2.7 (0.3)	2.6 (0.3)
LA/LV ratio	0.7 (1.0)	0.7 (0.1)	0.7 (0.1)	0.7 (0.1)
E/e' ratio	6.6 (1.7)	6.3 (1.4)	6.3 (1.7)	6.3 (1.6)
LV ejection fraction, %	70.8 (7.2)	70.8 (7.2)	71.2 (7.2)	70.9 (7.2)
LV ejection fraction <40%, % (*n*)	0.0 (0)	0.0 (0)	0.6 (1)	0.2 (1)

*Numbers are mean ±standard deviation or percentage and n. PA, physical activity, LDL, low-density lipoprotein, LV, left ventricular, LA, left atrial*.

Overall, there was a significant difference in increase in LADi (*p* = 0.018) and LVMi (*p* = 0.037) between groups of cumulative PA in multivariate adjusted analyses (Supplementary Table S3). No significant differences were observed between cumulative PA and change in the other echocardiography variables (LVDi, relative wall thickness, E/e' ratio, e' velocity, LV EF, and LA/LV ratio) (Supplementary Table S3).

### Cumulative PA and Change in LADi From Tromsø6 to Tromsø7

Overall, from Tromsø6 to Tromsø7, LADi increased significantly more in Hard compared to Moderate PA, with a mean group difference in LADi enlargement of 0.08 cm/m^2^ (95% CI 0.01–0.15, *p* = 0.020) (Table 2). No significant differences in LADi change were observed between Hard and Low PA (*p* = 0.128) and Moderate and Low PA (*p* = 1.000).

**Table 2:** Longitudinal associations between cumulative physical activity and change in left atrial diameter index: The Tromsø Study 2007–2016.

	**(*n*)**	**Model 1,** **baseline** **(mean ±SE)**	**Model 1,** **change** **(Delta ±95% CI)**	**Model 1 (*p*-value)**	**Model 2,** **baseline** **(Adjusted mean ±SE)**	**Model 2,** **change** **(Delta ±95% CI)**	**Model 2 (*p*-value)**
Total	572			0.011*			0.018*
Low PA	133	1.98 (0.02)	0.19 (0.14, 0.24)	Ref.	2.00 (0.02)	0.18 (0.13, 0.24)	Ref.
Moderate PA	249	1.98 (0.02)	0.17 (0.13, 0.21)	1.000	2.01 (0.02)	0.17 (0.13, 0.22)	1.000
Hard PA	190	2.02 (0.02)	0.26 (0.21, 0.30)	0.139	2.06 (0.02)	0.26 (0.21, 0.30)	0.128
Hard vs. Moderate				0.010			0.020
Males	258			<0.001*			<0.001*
Low PA	54	1.94 (0.04)	0.20 (0.12, 0.28)	Ref.	1.96 (0.04)	0.18 (0.10, 0.27)	Ref.
Moderate PA	91	1.97 (0.03)	0.13 (0.07, 0.19)	0.529	2.00 (0.03)	0.14 (0.08, 0.21)	1.000
Hard PA	113	2.00 (0.02)	0.30 (0.25, 0.35)	0.117	2.02 (0.03)	0.30 (0.24, 0.36)	0.047
Hard vs. Moderate				<0.001			<0.001
Females	314			0.950*			0.852*
Low PA	79	2.01 (0.03)	0.18 (0.11, 0.25)	Ref.	2.03 (0.03)	0.18 (0.10, 0.25)	Ref.
Moderate PA	158	1.99 (0.02)	0.19 (0.14, 0.24)	1.000	2.03 (0.02)	0.20 (0.14, 0.26)	1.000
Hard PA	77	2.05 (0.03)	0.19 (0.12, 0.26)	1.000	2.10 (0.03)	0.21 (0.13, 0.28)	1.000
Hard vs. Moderate				1.000			1.000
<65 years	363			0.015*			0.031*
Low PA	80	1.93 (0.03)	0.17 (0.11, 0.24)	Ref.	1.95 (0.03)	0.15 (0.08, 0.22)	Ref.
Moderate PA	169	1.98 (0.02)	0.13 (0.09, 0.18)	1.000	2.00 (0.02)	0.11 (0.06, 0.17)	1.000
Hard PA	114	1.98 (0.02)	0.24 (0.18, 0.29)	0.388	2.02 (0.03)	0.21 (0.15, 0.27)	0.474
Hard vs. Moderate				0.012			0.025
≥65 years	209			0.420*			0.262*
Low PA	53	2.06 (0.04)	0.21 (0.13, 0.30)	Ref.	2.06 (0.04)	0.23 (0.14, 0.32)	Ref.
Moderate PA	80	2.00 (0.03)	0.25 (0.18, 0.32)	1.000	2.00 (0.03)	0.28 (0.20, 0.26)	1.000
Hard PA	76	2.09 (0.03)	0.29 (0.21, 0.36)	0.585	2.10 (0.03)	0.33 (0.24, 0.41)	0.312
Hard vs. Moderate				1.000			1.000

* *p-value for main effect. Left atrial diameter index (LADi) and delta are presented as cm/m^2^. Model 1, unadjusted; Model 2, age, sex, body mass index, hypertension groups; PA; physical activity; LA, left atrial; SE, standard error; CI, confidence interval*.

In sex-stratified adjusted analysis (Table 2), LADi in males increased significantly more in Hard (0.30 cm/m^2^, SE 0.03) than in Moderate (0.14 cm/m^2^, SE 0.03) and Low PA (0.18 cm/m^2^, SE 0.04), with a mean group difference of 0.12 cm/m^2^ (95% CI 0.00–0.24, *p* = 0.047) between Hard and Low, and a mean group difference of 0.16 cm/m^2^ (95% CI 0.06–0.26, *p* < 0.001) between Hard and Moderate PA. No statistical difference between Moderate and Low PA was observed (*p* = 1.000). In females, no differences were observed (*p* = 0.852).

In age-stratified adjusted analysis (Table 2), LADi in participants <65 years increased significantly more in Hard (0.21 cm/m2, SE 0.03) than in Moderate (0.11 cm/m^2^, SE 0.03) PA, with a mean group difference of 0.10 cm/m^2^ (95% CI 0.01–0.18, *p* = 0.025). No statistical difference between Hard and Low PA (*p* = 0.474), or between Moderate and Low PA (*p* = 1.000) was observed. No statistical differences in LADi between groups were observed for participants ≥65 years (*p* = 0.262).

**Figure 3:**
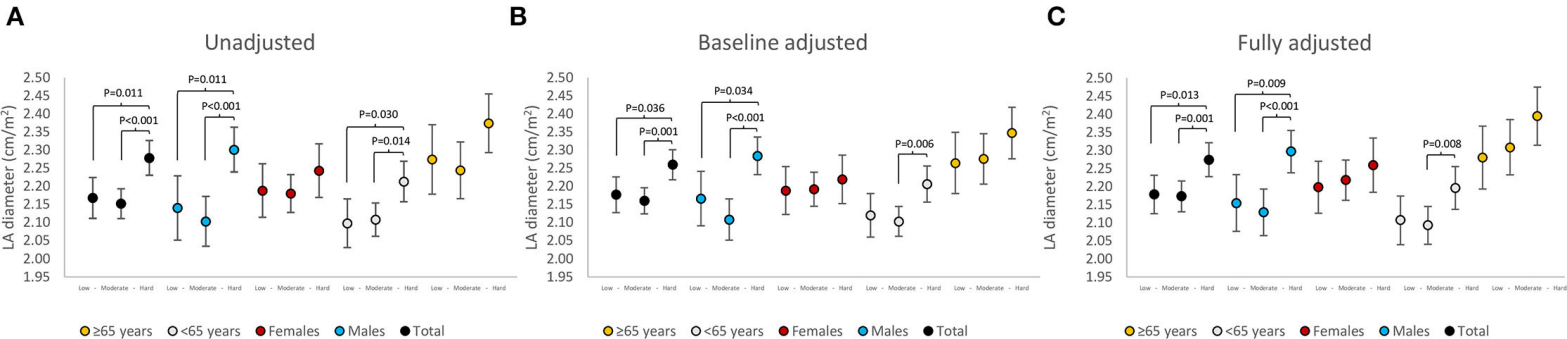
Associations between cumulative PA and cross-sectional LA diameter index (LADi) in Tromsø7. **(A)** is unadjusted, **(B)** is adjusted for baseline LADi in Tromsø6, **(C)** is adjusted for baseline LADi in addition to age, sex, body mass index, and hypertension groups. Differences between groups of cumulative PA are indicated with *p*-values. Error bars indicate 95% CI. PA, physical activity; LA, left atrial; CI, confidence interval.

**Figure 4:**
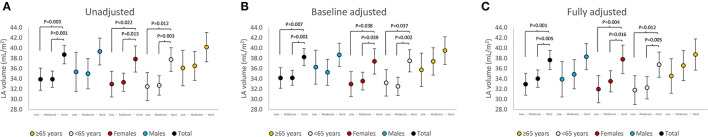
Associations between cumulative PA and cross-sectional LA volume index (LAVi) in Tromsø7. **(A)** is unadjusted, **(B)** is adjusted for baseline LADi in Tromsø6, **(C)** is adjusted for baseline LADi in addition to age, sex, body mass index, and hypertension groups. Differences between groups of cumulative PA is indicated with *p*-values. Error bars indicate 95% CI. PA, physical activity; LA, left atrial; CI, confidence interval; LADi, LA diameter index.

Associations between cumulative PA and cross-sectional LADi in Tromsø7 are presented in Figure 3. Significant associations between cumulative PA and LADi were observed in males, in participants <65 years, and in the overall analysis. Moreover, similar trends in the association between cumulative PA and LADi were observed in unadjusted, baseline adjusted, and in fully adjusted analysis. Associations between cumulative PA and cross-sectional LA volume index in Tromsø7 are presented in Figure 4. Significant associations between cumulative PA and LA volume index were observed in females, in participants <65 years, and in the overall analysis. Moreover, similar trends in the association between cumulative PA and LA volume index were observed in unadjusted, baseline adjusted, and in fully adjusted analysis.

### Cumulative PA and Change in LVMi From Tromsø6 to Tromsø7

Overall, from Tromsø6 to Tromsø7, LVMi increased significantly more in Moderate compared to Low PA, with a mean group difference in LVMi enlargement of 3.9 g/m^2.7^ (95% CI 0.23–7.57, *p* = 0.037) (Table 3). No significant differences in LVMi change were observed between Hard and Low PA (*p* = 0.172) and Hard and Moderate PA (*p* = 1.000).

**Table 3:** Longitudinal associations between cumulative physical activity and change in left ventricular mass index from baseline: The Tromsø Study 2007–2016.

	**(*n*)**	**Model 1,** **baseline** **(Mean ±SE)**	**Model 1,** **change** **(Delta ±95% CI)**	**Model 1 (*p*-value)**	**Model 2,** **baseline** **(Adjusted mean ±SE)**	**Model 2,** **change** **(Delta ±95% CI)**	**Model 2 (*p*-value)**
Total	494			0.077*			0.037*
Low PA	109	42.0 (1.0)	2.91 (0.53, 5.30)	Ref.	40.8 (0.9)	2.02 (−0.67, 4.70)	Ref.
Moderate PA	218	37.1 (0.7)	6.29 (4.60, 7.97)	0.071	38.8 (0.7)	5.92 (3.84, 8.00)	0.033
Hard PA	167	40.3 (0.8)	5.28 (3.35, 7.21)	0.391	41.1 (0.8)	5.10 (2.88, 7.33)	0.172
Hard vs. Moderate				1.000			1.000
Males	215			0.335*			0.224*
Low PA	43	45.1 (1.6)	3.46 (−0.93, 7.84)	Ref.	42.4 (1.6)	3.31 (−1.53, 8.15)	Ref.
Moderate PA	78	40.9 (1.2)	3.97 (0.71, 7.23)	1.000	41.7 (1.2)	3.86 (0.04, 7.68)	1.000
Hard PA	94	43.2 (1.1)	6.74 (3.77, 9.70)	0.670	44.3 (1.1)	7.25 (3.79, 10.72)	0.516
Hard vs. Moderate				0.651			0.412
Females	279			0.002*			0.001^*§^
Low PA	66	40.0 (1.1)	2.56 (−0.08, 5.19)	Ref.	38.9 (1.1)	0.86 (−2.17, 3.89)	Ref.
Moderate PA	140	35.0 (0.8)	7.58 (5.77, 9.39)	0.007	36.4 (0.9)	6.43 (4.09, 8.77)	0.002
Hard PA	73	36.6 (1.1)	3.40 (0.89, 5.91)	1.000	38.3 (1.0)	2.30 (−0.56, 5.15)	1.000
Hard vs. Moderate				0.025			0.027
<65 years	339			0.431*			0.182^*§^
Low PA	71	40.3 (1.1)	4.91 (2.44, 7.38)	Ref.	40.4 (1.1)	3.56 (0.67, 6.46)	Ref.
Moderate PA	158	36.2 (0.7)	6.84 (5.19, 8.50)	0.604	38.5 (0.8)	6.39 (4.23, 8.56)	0.196
Hard PA	110	37.3 (0.9)	5.97 (3.99, 7.96)	1.000	39.0 (0.9)	5.67 (3.34, 8.00)	0.616
Hard vs. Moderate				1.000			1.000
≥65 years	155			0.223*			0.204*
Low PA	38	45.1 (1.8)	−0.81 (−6.02, 4.40)	Ref.	42.9 (1.7)	−0.66 (−6.31, 4.99)	Ref.
Moderate PA	60	39.5 (1.5)	4.83 (0.68, 8.98)	0.289	40.0 (1.5)	5.20 (0.60, 10.44)	0.255
Hard PA	57	46.2 (1.5)	3.94 (−0.31, 8.20)	0.494	45.9 (1.6)	4.71 (−0.51, 9.93)	0.459
Hard vs. Moderate				1.000			1.000

* *p-value for main effect*.

§ *Assumption of equality of error variances is violated*.

*Left ventricular mass index (LVMi) and delta are presented as g/m^2.7^*.

*Model 1, unadjusted; Model 2, age, sex, body mass index, hypertension groups; PA, physical activity; LV, left ventricular; SE, standard error; CI, confidence interval*.

In sex-stratified adjusted analysis (Table 3), LVMi in females increased significantly more in Moderate than in Low PA, with a mean difference in change of 5.6 g/m^2.7^ (95% CI 1.61–9.53, *p* = 0.002), and in Moderate than Hard PA, with a mean difference in LVMi enlargement of 4.1 g/m^2.7^ (95% CI 0.35–7.92, *p* = 0.027). No significant difference between Hard and Low PA (p=1.000) was observed. In males, no significant differences were observed (*p* = 0.224). In age-stratified adjusted analysis (Table 3), no significant differences were observed (*p* ≥ 0.182).

In analysis of the association between cumulative PA and change in LVDi, significant associations were observed in females only (Supplementary Table S4). LVDi increased significant more in Moderate (0.07 cm/m^2^ ± SE 0.03) than in Low PA (−0.06 cm/m^2^, SE 0.04), with a mean group difference of 0.13 cm/m^2^ (95% CI 0.03 to 0.24, *p* = 0.010). However, no significant differences in LVDi change were observed between Hard and Low PA (*p* = 0.087) and Hard and Moderate PA (*p* = 1.000).

**Figure 5:**
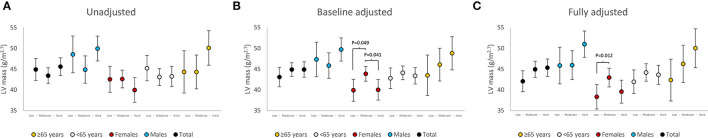
Associations between cumulative PA and cross-sectional LV mass index (LVMi) in Tromsø7. **(A)** is unadjusted, **(B)** is adjusted for baseline LVMi in Tromsø6, **(C)** is adjusted for baseline LVMi in addition to age, sex, body mass index, and hypertension groups. Differences between groups of cumulative PA are indicated with *p*-values. Error bars indicate 95% CI. PA, physical activity; LV, left ventricle; CI, confidence interval.

Associations between cumulative PA and cross-sectional LVMi in Tromsø7 are presented in Figure 5. Significant associations between cumulative PA and LVDi were observed in females only. Moreover, similar trends in the association between cumulative PA and LVMi were observed in unadjusted, baseline adjusted, and in fully adjusted analysis.

### Cumulative PA and Change in Cardiac Function From Tromsø6 to Tromsø7

The observed association observed between cumulative PA and change in LA/LV ratio overall (*p* = 0.075), stratified by sex (*p* ≥ 0.065), or stratified by age (*p* ≥ 0.083) was non-significant (Supplementary Table S5). No significant association was observed between cumulative PA and change in Mitral annular e' velocity overall (*p* = 0.903), stratified by sex (*p* ≥ 0.529), stratified by age (*p* ≥ 0.749), or stratified by hypertension (*p* ≥ 0.196) (Supplementary Table S6). No significant association was observed between cumulative PA and change in change in E/e' ratio overall (*p* = 0.253), stratified by sex (*p* ≥ 0.286), stratified by age (*p* ≥ 0.213), or stratified by body mass index (*p* ≥ 0.180) (Supplementary Table S7). No significant association was observed between cumulative PA and change in LV EF overall (*p* = 0.970), stratified by sex (*p* ≥ 0.652), or stratified by age (*p* ≥ 0.556) (Supplementary Table S8).

### Sensitivity Analysis

When we stepwise, or jointly, excluded participants with known cardiac pathologies, we observed rather similar associations between cumulative PA and change in echocardiography measurements as in the fully adjusted model 2 (Supplementary Table S9). Furthermore, when smoking, alcohol consumption, diabetes, and LDL cholesterol were stepwise added to model 2, the associations between cumulative PA and change in LADi and between cumulative PA and change in LVMi did not change, except when diabetes was added to the model, the association between cumulative PA and LVMi was no longer significant (*p* = 0.068).

## Discussion

Exercise-induced cardiac remodeling is well documented in athletes. Our longitudinal study adds to this knowledge by showing that more moderate levels of habitual PA over time is associated with cardiac remodeling also in a general adult and elderly population. The main finding from our study is that higher levels of cumulative PA was associated with increased LA size in males and in participants <65 years, and that moderate level of cumulative PA was associated with increased LV size in females.

Despite the association between cumulative PA and increased LA and LV sizes, indices of cardiac pump function and atrioventricular remodeling did not differ significantly between groups of cumulative PA. This may indicate that cardiac chamber enlargement is a physiological response to PA.

### Cumulative PA and Increased LA and LV Sizes

In our study of the general population, we observed that Hard cumulative PA was associated with a larger increase in LADi than lower PA levels; although when stratified by sex or age, increased LADi was only observed in males and in participants <65 years. Furthermore, Moderate cumulative PA was associated with increased LVMi and LVDi, but only in females when stratified by sex or age.

Our observations are at large consistent with previous studies of athletes. Several meta-analyses have reported that LA and LV sizes are larger in endurance trained athletes compared to non-athletes or sedentary controls (9, 30–32). Moreover, the relationship between endurance training and increased LV volume and mass has been demonstrated in a recent meta-analysis of both males and females (33). Additionally, the relationship between endurance training and increased LA volume, LV volume and LV mass has been confirmed in endurance trained young male and female athletes after 90 days of training (34). Similarly, increased LV volume and mass has been demonstrated in young sedentary males and females after 1 year of intensive endurance training (35).

Furthermore, the relationship between endurance training and enlarged LA volume and LV volume has been confirmed in middle-aged sedentary males and females after 10 months high-intensity endurance training (36); both LA and LV volumes increased significantly, but were considerably smaller when compared with a control group of age matched endurance athletes with a long history of endurance training (36). Additionally, a relationship between high levels of cumulative lifetime training hours and larger LA volume has been observed in males (37, 38), but no association between cumulative lifetime training hours and change in LV diameter or mass (38). Similarly, Mahjoub and colleagues demonstrated that LA volume increased after only 6 weeks of high-intensity endurance training in endurance trained men, whereas no change in LV mass, volume, or diameter was seen (39).

The findings from the previously discussed studies demonstrate that extensive LA and LV remodeling requires high amounts of endurance training stimuli over a long time. Similarly, our results indicate that the left atrium adapts faster to exercise than the left ventricle, and that sufficient and potentially higher stimulus from exercise intensity and volume is required for the left ventricle to remodel. The faster LA remodeling may be explained by the fact that the LA walls are thinner than the LV walls (11), and therefore are more affected by the hemodynamic overload during exercise according to the Laplace's law. In our study, the stimulus from moderate levels of habitual PA over time may have been insufficient to induce LV remodeling, which may explain the lack of association between cumulative PA and increased LV sizes in our study, except for females with Moderate PA. Furthermore, the lack of association between cumulative PA and change in LV size is supported by our sensitivity analysis, as the association between cumulative PA and change in LVMi became weaker and non-significant when we adjusted for diabetes.

### Age and Sex Modifications

It is well documented that the prevalence of cardiovascular risk factors such as hypertension increase progressively with age, with a prevalence of 60% in participants ≥60 years (21). Untreated hypertension causes LV hypertrophy, which impairs LV relaxation and induces LV diastolic dysfunction and LA enlargement (21). Thus, the lack of increase in LADi in participants ≥65 years may be due to age related changes in the heart.

The observed sex differences in the association between cumulative PA and LA size may be explained by physiological and morphological differences between males and females (40). Females are on average smaller, have lower lean mass and a different sex hormone profile than males, which significantly impacts cardiac size (41). As females generally have smaller cardiac chambers than males (19), males exhibit more pronounced cardiac changes despite similar relative increase in chamber sizes (41). Finally, there may be quantitative and qualitative differences in exercise patterns between males and females (40). This is supported by accelerometry-measured data from the general adult and elderly population, where females accumulated more minutes of light PA and males accumulated more minutes of moderate and vigorous PA (42, 43).

### Cumulative PA and Change in Cardiac Function

Despite the association between cumulative PA and increased LA and LV sizes, no significant differences in mitral annular e' velocity, E/e' ratio, LV EF, or LADi/LVDi ratio were observed between groups of cumulative PA. Thus, our results demonstrate that changes in indices of LV diastolic and systolic function, and atrioventricular chamber ratio, do not differ between groups of cumulative PA. The observed cardiac chamber enlargement with higher levels of cumulative PA in our study seems to be a physiological adaptation to exercise.

Our observations are consistent with previous reports of preserved cardiac function in athletes with exercise-induced cardiac remodeling. Studies of endurance athletes and elite soccer players have observed that LV diastolic function, as measured by Mitral valve and/or Tissue Doppler imaging, is preserved or even supranormal in athletes with cardiac chamber enlargement (13, 14, 38, 44). Also, studies have observed that LV systolic function, as measured by LV EF and/or LV fractional shortening, is normal in athletes with cardiac chamber enlargement (13, 15). Furthermore, preserved LV systolic function in athletes, as measured by LV EF and LV fractional shortening, has been confirmed in a meta-analysis of males and females at rest and during exercise (31). The authors observed no differences in LV systolic function between athletes and matched control subjects (31). Additionally, the effects of endurance training has been evaluated in a recent meta-analysis, where it was demonstrated that LV systolic function, as measured by LV EF and LV stroke volume, was slightly increased in males, but unaltered in females (33). In cross-sectional studies from the general population, no significant associations between increased LA volume and LV diastolic dysfunction, as measured by E/e' ratio, e', and/or tricuspid regurgitation velocity, was observed in physically active participants (45, 46).

In contrast, Lakatos and colleagues observed normal, but lower LV systolic function, as measured by LV EF and/or LV global longitudinal strain, in elite endurance athletes compared to non-athletes (47). Similarly, despite no difference in LV EF, it has been observed that LV global longitudinal strain was lower in elite endurance athletes than in non-athletes (13). With exercise-induced LV remodeling, it is possible that less myocardial deformation is required to obtain the same stroke volume. Therefore, reduced LV global longitudinal strain may be an adaptive change in elite endurance athletes (13).

Our observations of a balanced LADi/LVDi ratio, despite increased LA and LV sizes, is consistent with studies observing symmetrical enlargement of all four chambers (34), and that LA volume/LV volume ratio is similar despite LA enlargement in endurance athletes (13). An increased LA/LV ratio may be due to increased LV pressure and/or LV diastolic impairment, whereas an increased LA chamber with normal LA/LV ratio likely reflects a physiological adaptation to exercise (13).

In contrast to preserved pump function and balanced remodeling in the athlete's heart, increased risk of atrial fibrillation is seen in both adult and elderly endurance athletes (48, 49). It is suggested that LA enlargement itself may be a substrate for atrial fibrillation in athletes (50, 51), and therefore that the athlete's heart may potentially be proarrhythmic independent of other abnormalities. However, convincing data linking the combination of exercise and LA size to AF are lacking and are largely speculative (50, 52). Moreover, in a recent study investigating the acute effects of strenuous endurance exercise on atrial size and function in master athletes (53), the authors reported no exercise-induced atrial dysfunction or change in atrial size after an ultramarathon compared with baseline. Moreover, acute exercise-induced atrial fibrillation was uncommon during the race (53).

### Strengths and Limitations

The main strength of our study is the longitudinal design with repeated measurements of PA and echocardiographic structural and functional data, which enables evaluation of the direction of the associations as well as change from baseline. Moreover, the broad diversity of covariates allowed us to adjust for multiple potential confounders.

Our study has several limitations that should be addressed. First, due to the observational nature of this study, causation cannot be established. Second, LADi, LVDi, and LV EF were assessed by linear measurements, which are less accurate and have more geometrically assumptions than the recommended biplane volume calculated parameters (19). However, in Tromsø7, we found moderate correlation between biplane-calculated EF and Teichholz-calculated EF (*r* = 0.42), and between LADi and LA volume index (*r* = 0.45). Furthermore, LVDi correlated strongly with LV volume index (*r* = 0.87). Third, our study lacks assessment of LA function which may distinguish between pathological and physiological remodeling (8). Fourth, self-reported PA is prone to both recall- and social desirability bias (54), and misclassifications would probably underestimate the true effects of PA. However, in a sub-study of Tromsø6, self-reported PA using the Saltin-Grimby Physical Activity Level Scale was significantly correlated with maximal oxygen uptake (females *r*_s_ = 0.40, males *r*_s_ = 0.44, both *p* < 0.001) (55). Moreover, there was a significant positive linear trend between maximal oxygen uptake and levels of self-reported PA (55). This is consistent with the sensitivity analysis of cumulative PA performed on our analytical sample (Supplementary Table S2). Fifth, we cannot exclude residual confounding by measured or unmeasured variables (e.g. masked hypertension or sex hormones). Finally, the relatively low sample size in our study represents a potential limitation. However, baseline characteristics did not differ between our analytical sample and the total cohort attending the first visit in Tromsø6 (*n* = 12,981), which strengthens our external validity to other Northern-European Caucasian adult populations.

In conclusion, higher levels of cumulative PA were associated with increased LADi in males and participants <65 years, and with increased LVMi and LVDi in females. Despite the association between cumulative PA and cardiac chamber enlargement, the function of the heart did not change with higher levels of PA, and the atrioventricular ratio was unchanged. This indicate that cardiac chamber enlargement is a physiological response to PA.

## Data Availability Statement

The datasets presented in this article are not readily available because the legal restriction on data availability is set by the Tromsø Study Data and Publication Committee to control for data sharing, including publication of datasets with the potential of reverse identification of deidentified sensitive participant information. The data can however be made available from the Tromsø Study upon application to the Tromsø Study Data and Publication Committee. Requests to access the datasets should be directed to the Tromsø Study, Department of Community Medicine, Faculty of Health Sciences, UiT The Arctic University of Norway; e-mail: tromsous@uit.no.

## Ethics Statement

The studies involving human participants were reviewed and approved by Regional Committee for Medical and Health Research Ethics, Tromsø, Norway (20828/REK Nord). The participants provided their written informed consent to participate in this study.

## Author Contributions

KH, BW, and BM contributed to conception or design of the work. KH drafted the manuscript. All authors contributed to acquisition or analysis of the data. All authors contributed to interpretation of the data, critically revised the manuscript, gave final approval, and agree to be accountable for all aspects of work ensuring integrity and accuracy.

## Funding

KH was supported by the Northern Norway Regional Health Authority (Grant Number HNF1406-18).

## Conflict of Interest

The authors declare that the research was conducted in the absence of any commercial or financial relationships that could be construed as a potential conflict of interest.

## Publisher's Note

All claims expressed in this article are solely those of the authors and do not necessarily represent those of their affiliated organizations, or those of the publisher, the editors and the reviewers. Any product that may be evaluated in this article, or claim that may be made by its manufacturer, is not guaranteed or endorsed by the publisher.
