# Supplementary material for: Longitudinal Associations Between Cumulative Physical Activity and Change in Structure and Function of the Left Side of the Heart: The Tromsø Study 2007–2016
Source: Front Cardiovasc Med. 2022 May 12;9:882077. doi: 10.3389/fcvm.2022.882077 (PMC9133513; doi:10.3389/fcvm.2022.882077)
Supplement: Supplementary file 1 [file Data_Sheet_1.pdf]

SUPPLEMENTAL MATERIAL

Supplemental Table S1. Sum of ranked PA in Tromsø6 and Tromsø7 stratified by level of cumulative PA: The Tromsø Study 2007-2016.

Cumulative PA	n	Tromsø 6 (mean score ±SD)	Tromsø 7 (mean score ±SD)	Change (Delta ±95% CI)^A	P-diff
Overall	594	2.0 (0.6)	2.1 (0.7)	0.09 (0.04, 0.15)	0.001
Low	140	1.3 (0.5)	1.3 (0.5)	0.03 (-0.11, 0.16)	0.678
Moderate	259	2.0 (0.2)	2.0 (0.2)	0.03 (-0.02, 0.08)	0.249
Hard	195	2.6 (0.6)	2.8 (0.5)	0.23 (0.10, 0.34)	<0.001

Numbers are mean ±standard deviation or change ±95% confidence intervals. PA: physical activity, SD: standard deviation, CI: confidence interval. ^{A}=difference in mean score between Tromsø7 and Tromsø6.

Supplemental Table S2. Accelerometry measured PA stratified by level of cumulative PA: The Tromsø Study 2007-2016.

Cumulative PA	N=426	Total PA (counts/min)	MVPA (min/day)	Steps/day
Low (2+3)	101	405.0 (145.6)	20.3 (20.1)	4690.1 (2195.5)
Moderate (4)	179	509.1 (154.5)	35.8 (25.3)	6510.2 (2593.1)
Hard (5+6+7+8)	146	570.3 (171.1)	48.3 (30.7)	7598.4 (2901.1)
p-trend		<0.001	<0.001	<0.001

Numbers are mean ±standard deviation. PA: physical activity.

Supplemental Table S3. Longitudinal associations between cumulative PA and change in heart structure and function: The Tromsø Study 2007-2016.

	Model 2 (n)	Model 2 (p-value)
LADi (cm/m^2)	572	0.018
LVDi (cm/m^2)	524	0.071
LVMi (g/h$^{2.7}$)	494	0.037
Relative wall thickness (cm)	525	0.319
E/e' ratio	535	0.253 §
TDI e' (cm/s)	536	0.903
LV ejection fraction (%)	523	0.970
LA/LV ratio	515	0.075

§=Assumption of equality of error variances is violated. In the model: age, sex, body mass index, hypertension groups. PA: physical activity, LADi: left atrial diameter index, LVDi: left ventricular diameter index, LVMi: left ventricular mass index, TDI: tissue Doppler imaging.

Supplemental Table S4. Longitudinal associations between cumulative PA and change in LV diameter index (LVDi) from baseline: The Tromsø Study 2007-2016.

	(n)	**Model 1, baseline** (Mean ±SE)	**Model 1, change** (Delta ±95% CI)	**Model 1** (p-value)	**Model 2, baseline** (Adjusted mean ±SE)	**Model 2, change** (Delta ±95% CI)	**Model 2** (p-value)
**Total**	524			0.155 *			0.071 *
Low PA	119	2.71 (0.03)	0.01 (-0.04, 0.07)	Ref.	2.72 (0.03)	0.00 (-0.06, 0.07)	Ref.
Moderate PA	228	2.74 (0.02)	0.06 (0.01, 0.10)	0.744	2.71 (0.02)	0.07 (0.02, 0.12)	0.190
Hard PA	177	2.76 (0.02)	0.09 (0.04, 0.14)	0.162	2.76 (0.02)	0.09 (0.04, 0.14)	0.078
Hard vs Moderate				0.982			1.000
**Males**	234			0.057 *			0.211 *
Low PA	47	2.56 (0.04)	0.10 (0.01, 0.20)	Ref.	2.61 (0.04)	0.07 (-0.04, 0.17)	Ref.
Moderate PA	84	2.62 (0.03)	0.05 (-0.03, 0.12)	1.000	2.61 (0.03)	0.06 (-0.02, 0.14)	1.000
Hard PA	103	2.72 (0.03)	0.12 (0.06, 0.19)	1.000	2.69 (0.03)	0.14 (0.07, 0.21)	0.735
Hard vs Moderate				0.423			0.292
**Females**	290			0.328 *			0.011 *
Low PA	72	2.81 (0.03)	-0.04 (-0.12, 0.03)	Ref.	2.83 (0.04)	-0.06 (-0.14, 0.02)	Ref.
Moderate PA	144	2.82 (0.02)	0.06 (0.01, 0.11)	0.054	2.81 (0.03)	0.07 (0.01, 0.13)	0.010
Hard PA	74	2.84 (0.03)	0.04 (-0.03, 0.11)	0.293	2.82 (0.03)	0.05 (-0.03, 0.13)	0.087
Hard vs Moderate				1.000			1.000
**<65 years**	350			0.301 *			0.156 *
Low PA	74	2.68 (0.03)	0.03 (-0.04, 0.10)	Ref.	2.71 (0.03)	0.03 (-0.05, 0.11)	Ref.
Moderate PA	162	2.75 (0.02)	0.07 (0.03, 0.12)	1.000	2.72 (0.02)	0.10 (0.04, 0.16)	0.288
Hard PA	114	2.74 (0.03)	0.10 (0.05, 0.16)	0.367	2.73 (0.03)	0.11 (0.05, 0.17)	0.208
Hard vs Moderate				1.000			1.000
**≥65 years**	174			0.531 *			0.339 *
Low PA	45	2.76 (0.04)	-0.02 (-0.12, 0.09)	Ref.	2.76 (0.04)	-0.06 (-0.17, 0.05)	Ref.
Moderate PA	66	2.72 (0.04)	0.02 (-0.07, 0.10)	1.000	2.70 (0.04)	0.00 (-0.10, 0.10)	1.000
Hard PA	63	2.80 (0.04)	0.06 (-0.03, 0.15)	0.809	2.83 (0.04)	0.05 (-0.05, 0.16)	0.427
Hard vs Moderate				1.000			1.000

*=p-value for main effect. LVDi and delta are presented as cm/m^2. Model 1: unadjusted. Model 2: age, sex, BMI, hypertension groups. PA: physical activity, LV: left ventricular, PA: physical activity, SE: standard error, CI: confidence interval.

Supplemental Table S5. Longitudinal associations between cumulative PA and change in LA/LV ratio: The Tromsø Study 2007-2016.

	(n)	**Model 1, baseline** (Mean ±SE)	**Model 1, change** (Delta ±95% CI)	**Model 1** (p-value)	**Model 2, baseline** (Adjusted mean ±SE)	**Model 2, change** (Delta ±95% CI)	**Model 2** (p-value)
**Total**	515			0.146 *			0.075 *
Low PA	117	0.73 (0.01)	0.07 (0.05, 0.10)	Ref.	0.73 (0.01)	0.07 (0.05, 0.10)	Ref.
Moderate PA	225	0.73 (0.01)	0.05 (0.03, 0.06)	0.319	0.75 (0.01)	0.04 (0.02, 0.06)	0.161
Hard PA	173	0.73 (0.01)	0.07 (0.05, 0.09)	1.000	0.74 (0.01)	0.07 (0.04, 0.09)	1.000
Hard vs Moderate				0.296			0.196
**Males**	232			0.071 *			0.100 *
Low PA	47	0.76 (0.01)	0.04 (0.00, 0.08)	Ref.	0.75 (0.02)	0.04 (-0.00, 0.08)	Ref.
Moderate PA	83	0.76 (0.01)	0.04 (0.01, 0.07)	1.000	0.77 (0.01)	0.03 (0.00, 0.07)	1.000
Hard PA	102	0.74 (0.01)	0.08 (0.05, 0.11)	0.352	0.74 (0.01)	0.08 (0.05, 0.11)	0.491
Hard vs Moderate				0.094			0.129
**Females**	283			0.111 *			0.065 *
Low PA	70	0.71 (0.01)	0.09 (0.06, 0.12)	Ref.	0.71 (0.01)	0.10 (0.06, 0.14)	Ref.
Moderate PA	142	0.71 (0.01)	0.05 (0.03, 0.08)	0.142	0.73 (0.01)	0.05 (0.03, 0.08)	0.072
Hard PA	71	0.72 (0.01)	0.05 (0.02, 0.09)	0.266	0.74 (0.01)	0.06 (0.02, 0.09)	0.198
Hard vs Moderate				1.000			1.000
**<65 years**	341			0.177 *			0.083 *
Low PA	72	0.72 (0.01)	0.05 (0.02, 0.08)	Ref.	0.72 (0.01)	0.04 (0.00, 0.07)	Ref.
Moderate PA	159	0.72 (0.01)	0.03 (0.01, 0.05)	0.968	0.74 (0.01)	0.01 (-0.01, 0.04)	0.619
Hard PA	110	0.72 (0.01)	0.06 (0.04, 0.09)	1.000	0.73 (0.01)	0.05 (0.02, 0.08)	1.000
Hard vs Moderate				0.204			0.094
**≥65 years**	174			0.641 *			0.554 *
Low PA	45	0.74 (0.02)	0.11 (0.06, 0.15)	Ref.	0.74 (0.02)	0.13 (0.08, 0.18)	Ref.
Moderate PA	66	0.75 (0.01)	0.08 (0.04, 0.12)	1.000	0.76 (0.02)	0.10 (0.06, 0.14)	0.909
Hard PA	63	0.75 (0.01)	0.08 (0.04, 0.12)	1.000	0.75 (0.02)	0.10 (0.06, 0.15)	1.000
Hard vs Moderate				1.000			1.000

*=p-value for main effect. LA/LV ratio is presented as LA diameter/LV diameter. Model 1: unadjusted. Model 2: age, sex, body mass index, hypertension groups. PA: physical activity, LA: left atrial, LV: left ventricular, PA: physical activity, SE: standard error, CI: confidence interval.

Supplemental Table S6. Longitudinal associations between cumulative PA and change in average TDI e' velocity: The Tromsø Study 2007-2016.

	(n)	**Model 1, baseline** (Mean ±SE)	**Model 1, change** (Delta ±95% CI)	**Model 1** (p-value)	**Model 2, baseline** (Adjusted mean ±SE)	**Model 2, change** (Delta ±95% CI)	**Model 2** (p-value)
**Total**	536			0.788 *			0.903 *
Low PA	123	11.2 (0.2)	-3.7 (-4.02, -3.33)	Ref.	11.4 (0.2)	-3.8 (-4.19, -3.44)	Ref.
Moderate PA	238	12.0 (0.2)	-3.8 (-4.06, -3.56)	1.000	11.8 (0.1)	-3.8 (-4.13, -3.54)	1.000
Hard PA	175	12.0 (0.2)	-3.7 (-4.00, -3.43)	1.000	11.8 (0.2)	-3.7 (-4.07, -3.42)	1.000
Hard vs Moderate				1.000			1.000
**Normotensive**[A]	260			0.228 *			0.196 *
Low PA	51	12.3 (0.3)	-4.3 (-4.83, -3.77)	Ref.	12.8 (0.3)	-4.3 (-4.88, -3.79)	Ref.
Moderate PA	117	12.9 (0.2)	-3.8 (-4.12, -3.42)	0.289	12.8 (0.2)	-3.8 (-4.12, -3.39)	0.241
Hard PA	92	13.0 (0.2)	-3.8 (-4.22, -3.43)	0.454	12.9 (0.2)	-3.8 (-4.21, -3.42)	0.400
Hard vs Moderate				1.000			1.000
**Hypertensive**[B]	276			0.096 *			0.261 *
Low PA	72	10.4 (0.2)	-3.2 (-3.67, -2.77)	Ref.	10.5 (0.2)	-3.3 (-3.78, -2.87)	Ref.
Moderate PA	121	11.2 (0.2)	-3.9 (-4.20, -3.50)	0.092	11.1 (0.2)	-3.8 (-4.14, -3.45)	0.288
Hard PA	83	10.9 (0.2)	-3.6 (-4.02, -3.18)	0.701	11.0 (0.2)	-3.6 (-4.02, -3.16)	1.000
Hard vs Moderate				1.000			1.000
**Males**	232			0.536 *			0.579 *
Low PA	47	10.9 (0.3)	-3.4 (-3.97, -2.77)	Ref.	10.9 (0.3)	-3.5 (-4.15, -2.85)	Ref.
Moderate PA	86	11.9 (0.2)	-3.8 (-4.22, -3.36)	0.853	11.7 (0.2)	-3.9 (-4.44, -3.41)	0.836
Hard PA	99	11.7 (0.2)	-3.7 (-4.14, -3.31)	1.000	11.7 (0.2)	-3.9 (-4.35, -3.40)	0.992
Hard vs Moderate				1.000			1.000
**Females**	304			0.858 *			0.529 *
Low PA	76	11.4 (0.3)	-3.9 (-4.27, -3.44)	Ref.	11.8 (0.2)	-4.0 (-4.43, -3.51)	Ref.
Moderate PA	152	12.1 (0.2)	-3.8 (-4.12, -3.53)	1.000	11.8 (0.2)	-3.8 (-4.17, -3.45)	1.000
Hard PA	76	12.4 (0.3)	-3.7 (-4.12, -3.29)	1.000	11.8 (0.2)	-3.7 (-4.12, -3.19)	0.889
Hard vs Moderate				1.000			1.000
**<65 years**	347			0.540 *			0.749 *
Low PA	75	11.8 (0.3)	-3.6 (-4.08, -3.21)	Ref.	11.6 (0.3)	-3.7 (-4.24, -3.24)	Ref.
Moderate PA	164	12.7 (0.2)	-3.8 (-4.13, -3.54)	1.000	12.1 (0.2)	-3.8 (-4.17, -3.41)	1.000
Hard PA	108	13.0 (0.2)	-4.0 (-4.33, -3.60)	0.802	12.4 (0.2)	-3.9 (-4.37, -3.52)	1.000
Hard vs Moderate				1.000			1.000
**≥65 years**	189			0.989 *			0.957 *
Low PA	48	10.3 (0.2)	-3.7 (-4.27, -3.14)	Ref.	10.4 (0.2)	-3.8 (-4.41, -3.22)	Ref.
Moderate PA	74	10.6 (0.2)	-3.7 (-4.20, -3.29)	1.000	10.7 (0.2)	-3.8 (-4.36, -3.23)	1.000
Hard PA	67	10.4 (0.2)	-3.3 (-3.78, -2.83)	0.856	10.5 (0.2)	-3.4 (-3.95, -2.84)	0.871
Hard vs Moderate				0.579			0.589

*=p-value for main effect. 'e' velocity is presented as cm/sec. Model 1: unadjusted. Model 2: age, sex, body mass index, hypertension groups. TDI: tissue Doppler imaging, PA: physical activity, SE: standard error, CI: confidence interval.

[A:] Normotensive: systolic blood pressure <140 mmHg, diastolic blood pressure <90 mmHg, and no self-reported use of antihypertensives.

[B:] Hypertensive: systolic blood pressure ≥140 mmHg and/or diastolic blood pressure ≥90 mmHg and/or self-reported use of antihypertensives.

Supplemental Table S7. Longitudinal associations between cumulative PA and change in E/e' ratio: The Tromsø Study 2007-2016.

	(n)	**Model 1, baseline** (Mean ±SE)	**Model 1, change** (Delta ±95% CI)	**Model 1** (p-value)	**Model 2, baseline** (Adjusted mean ±SE)	**Model 2, change** (Delta ±95% CI)	**Model 2** (p-value)
**Total**	535			0.056 *			0.253 *
Low PA	123	6.5 (0.1)	2.4 (1.99, 2.90)	Ref.	6.3 (0.1)	2.4 (0.3)	Ref.
Moderate PA	237	6.2 (0.1)	1.9 (1.58, 2.23)	0.178	6.2 (0.1)	2.0 (0.2)	0.447
Hard PA	175	6.1 (0.1)	1.73 (1.35, 2.12)	0.058	6.2 (0.1)	1.9 (0.2)	0.372
Hard vs Moderate				1.000			1.000
**BMI <25**	186			0.169 *			0.180 *
Low PA	29	6.6 (0.3)	2.4 (1.50, 3.26)	Ref.	6.6 (0.3)	2.1 (1.05, 3.14)	Ref.
Moderate PA	86	6.1 (0.2)	1.7 (1.18, 2.21)	0.561	6.2 (0.3)	1.4 (0.57, 2.31)	0.614
Hard PA	71	6.0 (0.2)	1.2 (0.66, 1.78)	0.089	6.2 (0.2)	1.1 (0.29, 1.93)	0.194
Hard vs Moderate				0.651			1.000
**BMI ≥25**	349			0.388 *			0.623 *
Low PA	94	6.5 (0.2)	2.5 (1.93, 3.00)	Ref.	6.4 (0.2)	2.4 (1.83, 2.94)	Ref.
Moderate PA	151	6.3 (0.1)	2.0 (1.60, 2.45)	0.616	6.3 (0.1)	2.1 (1.61, 2.53)	1.000
Hard PA	104	6.2 (0.1)	2.1 (1.58, 2.60)	0.950	6.3 (0.1)	2.3 (1.74, 2.82)	1.000
Hard vs Moderate				1.000			1.000
**Males**	231			0.953 *			0.917 *
Low PA	47	6.4 (0.2)	1.7 (1.06, 2.38)	Ref.	6.3 (0.2)	1.6 (0.90, 2.35)	Ref.
Moderate PA	85	6.1 (0.2)	1.7 (1.20, 2.18)	1.000	6.1 (0.2)	1.8 (1.22, 2.38)	1.000
Hard PA	99	6.0 (0.2)	1.6 (1.16, 2.06)	1.000	5.9 (0.2)	1.7 (1.18, 2.25)	1.000
Hard vs Moderate				1.000			1.000
**Females**	304			0.043 *			0.286 *
Low PA	76	6.6 (0.2)	2.9 (2.27, 3.51)	Ref.	6.5 (0.2)	2.8 (2.17, 3.49)	Ref.
Moderate PA	152	6.3 (0.1)	2.0 (1.58, 2.46)	0.076	6.5 (0.1)	2.3 (1.75, 2.78)	0.395
Hard PA	76	6.3 (0.2)	1.9 (1.28, 2.52)	0.080	6.6 (0.2)	2.3 (1.61, 2.94)	0.606
Hard vs Moderate				1.000			1.000
**<65 years**	347			0.625 *			0.863 *
Low PA	75	6.3 (0.2)	1.9 (1.41, 2.34)	Ref.	6.2 (0.2)	1.9 (0.3)	Ref.
Moderate PA	164	6.0 (0.1)	1.7 (1.37, 1.99)	1.000	6.1 (0.1)	1.7 (0.2)	1.000
Hard PA	108	5.9 (0.1)	1.6 (1.19, 1.97)	1.000	6.1 (0.1)	1.7 (0.2)	1.000
Hard vs Moderate				1.000			1.000
**≥65 years**	188			0.093 *			0.213 *
Low PA	48	6.9 (0.2)	3.3 (2.40, 4.26)	Ref.	6.7 (0.2)	3.1 (2.18, 4.11)	Ref.
Moderate PA	73	6.7 (0.2)	2.4 (1.65, 3.16)	0.388	6.5 (0.2)	2.2 (1.39, 3.07)	0.385
Hard PA	67	6.6 (0.2)	2.0 (1.20, 2.77)	0.092	6.5 (0.2)	2.1 (1.23, 3.01)	0.320
Hard vs Moderate				1.000			1.000

*=p-value for main effect. E/e' ratio is presented as the ratio between peak E-wave velocity/peak annular average e' velocity. Model 1: unadjusted. Model 2: age, sex, body mass index, hypertension groups. PA: physical activity, SE: standard error, CI: confidence interval, BMI: body mass index.

Supplemental Table S8. Longitudinal associations between cumulative PA and change in LV ejection fraction: The Tromsø Study 2007-2016.

	(n)	**Model 1, baseline** (Mean ±SE)	**Model 1, change** (Delta ±95% CI)	**Model 1** (p-value)	**Model 2, baseline** (Adjusted mean ±SE)	**Model 2, change** (Delta ±95% CI)	**Model 2** (p-value)
**Total**	523			0.747 *			0.970 *
Low PA	119	70.8 (0.7)	-8.89 (-11.03, -6.75)	Ref.	70.4 (0.7)	-7.83 (1.19)	Ref.
Moderate PA	228	71.0 (0.5)	-7.95 (-9,50, -6.41)	1.000	70.6 (0.6)	-7.60 (0.93)	1.000
Hard PA	176	71.0 (0.5)	-7.93 (-9.69, -6.17)	1.000	71.0 (0.6)	-7.48 (1.01)	1.000
Hard vs Moderate				1.000			1.000
**Males**	234			0.997 *			0.682 *
Low PA	47	70.1 (1.1)	-8.45 (-11.97, -4.93)	Ref.	69.4 (1.2)	-6.37 (-10.01, -2.74)	Ref.
Moderate PA	85	69.8 (0.8)	-8.57 (-11.19, -5.95)	1.000	69.7 (0.9)	-8.25 (-11.10, -5.40)	1.000
Hard PA	102	69.8 (0.7)	-8.62 (-11.01, -6.23)	1.000	69.6 (0.8)	-8.07 (-10.68, -5.46)	1.000
Hard vs Moderate				1.000			1.000
**Females**	289			0.488 *			0.652 *
Low PA	72	71.3 (0.8)	-9.17 (-11.86, -6.48)	Ref.	71.5 (0.9)	-9.06 (-12.14, -5.98)	Ref.
Moderate PA	143	71.6 (0.6)	-7.59 (-9.49, -5.68)	1.000	71.9 (0.7)	-7.80 (-10.22, -5.38)	1.000
Hard PA	74	72.7 (0.8)	-6.97 (-9.62, -4.32)	0.756	73.0 (0.9)	-7.29 (-10.32, -4.27)	1.000
Hard vs Moderate				1.000			1.000
**<65 years**	348			0.475 *			0.556 *
Low PA	74	70.2 (0.8)	-7.47 (-9.94, -4.99)	Ref.	69.7 (1.0)	-6.79 (-9.67, -3.90)	Ref.
Moderate PA	161	70.6 (0.6)	-7.46 (-9.14, -5.78)	1.000	70.1 (0.7)	-7.03 (-9.19, -4.86)	1.000
Hard PA	113	69.6 (0.7)	-5.95 (-7.95, -3.95)	1.000	69.7 (0.8)	-5.57 (-7.93, -3.22)	1.000
Hard vs Moderate				0.768			0.866
**≥65 years**	175			0.566 *			0.679 *
Low PA	45	71.9 (1.0)	-11.23 (-15.19, -7.26)	Ref.	72.1 (1.1)	-11.02 (-15.21, -6.83)	Ref.
Moderate PA	67	71.8 (0.8)	-9.14 (-12.39, -5.89)	1.000	71.3 (0.9)	-9.26 (-12.99, -5.53)	1.000
Hard PA	63	73.5 (0.9)	-11.47 (-14.82, -8.12)	1.000	73.4 (1.0)	-11.24 (-15.18, -7.29)	1.000
Hard vs Moderate				0.974			1.000

*=p-value for main effect. LV ejection fraction is presented as %. Model 1: unadjusted. Model 2: age, sex, body mass index, hypertension groups. LV: left ventricular, PA: physical activity, SE: standard error, CI: confidence interval.

Supplemental Table S9. Longitudinal associations between cumulative PA and change in heart structure and function. Main effects with and without cardiac pathologies: The Tromsø Study 2007-2016.

	**TOTAL**		**Excluding AF (n=12)**		**Excluding MI (n=28)**		**Excluding stroke (n=11)**		**Excluding EF<40% (n=1)**		**Excluding cardiac pathologies [A] (n=46)**	
	Model 2 (n)	Model 2 (p-value)	Model 2 (n)	Model 2 (p-value)	Model 2 (n)	Model 2 (p-value)	Model 2 (n)	Model 2 (p-value)	Model 2 (n)	Model 2 (p-value)	Model 2 (n)	Model 2 (p-value)
LADi (cm/m^2)	572	0.018	560	0.026	539	0.004	555	0.013	516	0.023	472	0.010
LVDi (cm/m^2)	524	0.071	515	0.076	495	0.075	507	0.039	521	0.076	476	0.085
LVMi ($g/h^{2.7}$)	494	0.037	486	0.043	494	0.037	484	0.032	492	0.041	475	0.042
Relative wall thickness (cm)	525	0.319	516	0.387	496	0.279	508	0.279	522	0.293	477	0.384
E/e' ratio*	535	0.253 §	530	0.272	535	0.253 §	525	0.519	484	0.274 §	471	0.560
TDI e' (cm/s)	536	0.903	530	0.918	536	0.903	526	0.821	485	0.894	471	0.969
LV EF	523	0.970	514	0.929	494	0.965	506	0.866	522	0.971	477	0.796
LA/LV ratio	515	0.075	506	0.104	487	0.025	499	0.094	512	0.058	468	0.035

§=Assumption of equality of error variances is violated. In the model: age, sex, body mass index, hypertension groups. PA: physical activity, AF: atrial fibrillation, MI: myocardial infarction, LV EF: left ventricular ejection fraction, LADi: left atrial diameter index, LVDi: left ventricular diameter index, TDI: tissue Doppler imaging.

[A]Cardiac pathologies: AF, MI, stroke, and EF <40%.
